# Influence of Alpha-Actinin-3 R577X Polymorphism on Muscle Damage and the Inflammatory Response after an Acute Strength Training Session

**DOI:** 10.1155/2022/5447100

**Published:** 2022-12-16

**Authors:** Milton Amaral Pereira, Izinara Cruz Rosse, Ana Carolina Silva, Pedro José Fernandes Nunes Coelho, Bruno Magalhães de Castro, Lenice Kappes Becker, João Batista Ferreira Júnior, Emerson Cruz de Oliveira, Kelerson Mauro de Castro Pinto, André Talvani, Karina Barbosa de Queiroz, Daniel Barbosa Coelho

**Affiliations:** ^1^Health and Nutrition Postgraduate Program, Federal University of Ouro Preto (UFOP), Ouro Preto, Minas Gerais, Brazil; ^2^Department of Pharmacy, Federal University of Ouro Preto, Ouro Preto, Minas Gerais, Brazil; ^3^Program in Health and Nutrition, School of Physical Education, Exercise Physiology Laboratory, Federal University of Ouro Preto, Ouro Preto, Minas Gerais, Brazil; ^4^Department of Physical Education, Federal Institute of Sudeste of Minas Gerais, Rio Pomba, Minas Gerais, Brazil; ^5^School of Physical Education, Exercise Physiology Laboratory, Inflammation Immunobiology Laboratory, Federal University of Ouro Preto, Ouro Preto, Minas Gerais, Brazil; ^6^Department of Biological Sciences, Federal University of Ouro Preto, Health and Nutrition Program, Ouro Preto, Minas Gerais, Brazil; ^7^Department of Nutrition, Federal University of Ouro Preto, Health and Nutrition Postgraduate Program, Ouro Preto, Minas Gerais, Brazil

## Abstract

The objective of this study was to verify the influence of the ACTN3 R577X polymorphism on muscle damage and the inflammatory response after an acute strength training (ST) session. Twenty-seven healthy male individuals (age: 25 ± 4.3 years) participated in the study, including 18 RR/RX and 9 XX individuals. The participants were divided into two groups (RR/RX and XX groups) and subjected to an acute ST session, which consisted of a series of leg press, leg extension machine, and seated leg curl machine. The volunteers were instructed to perform the greatest volume of work until concentric muscle failure. Each volunteer's performance was analyzed as the load and total volume of training, and the blood concentrations of C-C motif chemokine ligand 2 (CCL2), interleukin-8 (IL-8), creatine kinase (CK), lactate dehydrogenase (LDH), myoglobin, testosterone, and cortisol were measured before the ST session and 30 min and 24 h postsession. The ACTN3 R577X polymorphism effect was observed, with increased concentrations of CCL2 (*p* < 0.01), IL-8 (*p* < 0.01), and LDH (*p* < 0.001) in XX individuals. There was an increase in the concentration of CK in the RR/RX group compared to XX at 24 h after training (*p* > 0.01). The testosterone/cortisol ratio increased more markedly in the XX group (*p* < 0.001). Regarding performance, the RR/RX group presented higher load and total volume values in the training exercises when compared to the XX group (*p* < 0.05). However, the XX group presented higher values of delayed onset muscle soreness (DOMS) than the RR/RX group (*p* < 0.05). The influence of ACTN3 R577X polymorphism on muscle damage and the inflammatory response was observed after an acute ST session, indicating that the RR/RX genotype shows more muscle damage and a catabolic profile due to a better performance in this activity, while the XX genotype shows more DOMS.

## 1. Introduction

Sports performance and muscle damage have been identified as the result of a combination of nutritional factors, training, psychological profile, and genetic predisposition. In this sense, studies showed that the genetic profile is a determining factor for success in certain sports [1–3]. Among the genes related to physical performance and muscle response, *ACTN3* stands out. In individuals with RR/RX genotypes for the ACTN3 R577X polymorphism, this gene encodes alpha-actinin sarcomeric protein-3, which is present in type II muscle fibers, that is responsible for playing an essential role in the generation force and power muscle contractions [4, 5].

The ACTN3 R577X (rs1815739) is a nonsense mutation that involves the replacement of an arginine (R) residue with a premature stop codon (X) at position 577 of the protein [4, 5]. Carriers of the R allele are generally associated with better performance in strength and power activities in elite athletes [6, 7], whereas the absence of alpha-actinin-3 in individuals with XX genotype has been associated with greater fitness for endurance activities and lower force generation capacity in animals and humans [8, 9]. These results, however, are divergent in studies with different groups and training protocols [10–12]. In addition, insufficient sampling also results in divergent results among the studies.

Although alpha-actinin-3 protein deficiency in individuals with XX genotype does not cause disease [13], this genotype has been characterized as being more susceptible to exertion-induced muscle injury, requiring a longer recovery period [14–16]. Studies that evaluated susceptibility to exercise-induced muscle damage by means of indirect and/or inflammatory markers suggest a greater vulnerability associated with genetic profile XX compared to genetic profiles RR and RX [14, 17–19].

The mechanism attributed to this greater susceptibility to muscle damage in the ACTN3-XX genotype is lower stability in the interaction between proteins in the Z-disc subjected to mechanical stress when compared to the ACTN3-RR/RX genotypes [20, 21]. On the other hand, it has been suggested that the expression of alpha-actinin-3 represents a protective mechanism against the development of lesions through an increase in type IIa muscle fiber stiffness [21].

Based on the above statements, genetic factors also may influence muscle strength production, muscle damage, and inflammatory response in strength training programs [22]. These exercise-induced physiological responses are classically measured by variations in the rate of creatine kinase (CK), interleukin-6 (IL-6), lactate dehydrogenase (LDH), myosin, troponin I, and myoglobin, which are markers of different muscle damage variations that physical demands may cause [23, 24]. However, the conclusions based on the analysis of these markers still need to be confirmed due to different exercise protocols and tested groups with distinct characteristics (age, gender, and previous training level) [25].

Furthermore, hormonal parameters can also show different responses based on the genetic profile after acute training sessions. The analyses of these hormonal parameters are useful to determine the testosterone and cortisol ratio (T : C ratio), which indicates a tendency to an anabolic or catabolic state. A catabolic condition previous to the training stimulus can be a sign of insufficient recovery from the previous stimulus or of some nutritional deficiency and can indicate overtraining [26, 27].

This seems to justify a trend observed in some observational studies in which ACTN3-XX athletes present a higher incidence of injuries and longer time away from work [15, 28, 29]. In this respect, it is believed that identifying physiological patterns in different genetic profiles through acute strength stimuli can provide information to optimize each athlete's physical conditioning, recovery, and progression, assisting in the prevention of injuries through the individualization of training.

Thus, the aim of the present study was to evaluate the influence of the ACTN3 R577X polymorphism on muscle damage and the inflammatory response after an acute strength training (ST) session.

## 2. Materials and Methods

### 2.1. Ethical Approval

This study was approved by the Research Ethics Committee of the Federal University of Ouro Preto (approval no. 79961317.0.0000.5150), following the standards established by the Brazilian National Health Council (resolution 466/2012). The volunteers were provided with information about the study and signed an informed consent form.

### 2.2. Participant Selection

Volunteer recruitment for this study was carried out with the goal of obtaining a minimum number of nine participants with the ACTN3-XX genotype, a group with lower genotypic frequency in the population. The sample size (*n* = 27) was calculated by comparing paired groups [30] considering a 95% confidence level and an 80% power.

Approximately 60 genotypic analyses were performed to reach the desired number of ACTN3-XX individuals. Overall, 27 healthy male individuals aged 25 ± 4.3 years and who had practiced weight training for at least six months participated in this study. The participants were divided into groups according to the alleles they carried for the ACTN3 gene. They were then classified as group RR/RX (*n* = 18) or group XX (*n* = 9).

Volunteers who did not show up on the scheduled day and time, who presented any illness that compromised the data collection, or who used any medication, nutritional supplements, or anabolic androgenic steroids were considered unsuitable for participation. The participants were instructed to keep their usual diets and not to perform vigorous physical activities before and during the period of the experiment.

### 2.3. Experimental Design

The subjects attended the laboratory on a total of three occasions in the morning period. The first visit consisted of an anthropometric evaluation: weight, height, body mass index (BMI) and fat percentage (%fat), a familiarization session with the study procedures, and a 10-repetition maximum (10RM) test.

In the familiarization session, two series were performed for each exercise, with volumes, intensities, and loads considered light to ensure that there was no muscular wasting that could influence the 10RM test [31]. The proposed exercises were leg press (LP), leg extension machine (LEM), and seated leg curl machine (SLCM). The session also involved the standardization of position, range of motion, and duration of concentric and eccentric muscle actions. Forty-eight hours after the first visit, both the RR/RX and XX groups performed a training session. Three blood collections were performed throughout the study to evaluate indirect markers of muscle damage: the first blood collection (C1) occurred before the training session, the second (C2) was performed 30 min after the end of the session, and the third (C3) was performed 24 h after the training session, during the third and last visit to the laboratory. The objective of the collections was to evaluate in the different periods the concentrations of C-C motif chemokine ligand 2 (CCL2), IL-8, CK, LDH, myoglobin, testosterone, and cortisol. The participants' performance regarding the load, volume, and total volume was also measured and recorded during the training session of each exercise. At the end of the experiment, the participants were also evaluated regarding their subjective perception of pain.

### 2.4. 10RM Test

A 10RM test was performed at the end of familiarization for each exercise to establish the load of the training session. The test protocol was adapted from Simão et al. [31] and followed the specifications below:

- Maximum number of five attempts at each exercise to determine the training load
- A 5 min pause between attempts
- Gradual progression of weight from the perception of volunteers and evaluators, based on a scale of subjective perception of effort from repetitions in reserve proposed by Helms et al. [32]

This procedure was adopted to maximize the test's level of specificity to the training session.

### 2.5. Strength Training Session

The ST session involved performing the following exercises in order: LP, LEM, and SLCM [33]. For each of the exercises, 4 sets of 10 maximum repetitions or the greatest number of repetitions were performed until concentric failure was reached [34] with a load of 85% of 1RM. The interval between the series and exercises was 90 s [35]. For standardization of the movements, the volunteers were instructed to follow a rhythm of 2 s for concentric actions and 4 s for eccentric actions at a 60 s cadence during the training session. In this way, the participant remained in eccentric action longer, aiming at causing greater muscle damage [36]. The number of repetitions and the weight lifted in each series were recorded. The training volume was calculated by multiplying the number of repetitions performed by the weight lifted.

### 2.6. Blood Collections and Indirect Markers of Muscle Damage

The measurement of muscle damage was performed by measuring physiological markers. The blood samples were collected by venipuncture in the volunteers' antecubital fossa which was performed by a trained nurse, respecting the biosafety standards recommended by the health authorities. In the first collection (C1), 12 mL of blood was collected, 7 mL in a no additive collection tube for laboratory marker determination and 5 mL in an EDTA tube for genotyping. In C2 and C3, only one 7 mL tube without anticoagulant was collected. The testosterone, cortisol, and myoglobin concentrations were analyzed by high selectivity chemiluminescence and affinity using Access Beckman Coulter® kits and equipment. The concentrations of CK, LDH, IL-8, and CCL2 were analyzed by the high-sensitivity enzyme-linked immunosorbent assay (ELISA) method (Sigma-Aldrich). To analyze the genetic profile of the participants, blood samples were collected using the vacuum method, in 2–4 mL EDTA tubes (Vacuette). To extract the genomic DNA from the peripheral blood, samples were performed according to the literature protocol, using proteinase K followed by salt precipitation [37]. Genotyping of the ACTN3 R577X polymorphism was performed as described by Coelho et al. [38].

### 2.7. Delayed Onset Muscle Soreness

The visual analog scale was used to infer the subjective perception of muscle pain 24 h after the training session. The participants were asked to rate their perceptions of pain on a 10 cm long numbered horizontal scale in which 0 (zero) indicated no pain and 10 the worst possible pain [39].

### 2.8. Statistical Analyses

Statistical analyses were performed using GraphPad Prism version 6.0 (GraphPad Software Inc., Irvine, CA). Data normality was tested using the Kolmogorov test. To verify the homogeneity of variances, the Levene test was used. The effect of SNP or period was compared using 2-way ANOVA followed by the Bonferroni post hoc test. Outliers were excluded before statistical analysis using the standard interquartile range (IQR) criteria (values above 75% and below 25%), when it was relevant. *p* values < 0.05 were considered statistically significant.

## 3. Results

The individuals were characterized according to their genetic profiles for ACTN3-R577X. There were 18 RR/RX and 9 XX individuals. The results obtained in the physical evaluation measuring the body composition, weight, height, and estimated body fat percentage are presented in Table 1. The homogeneity of variances for comparisons between groups was verified by the Levene test (*p* > 0.05).

**Figure 1:**
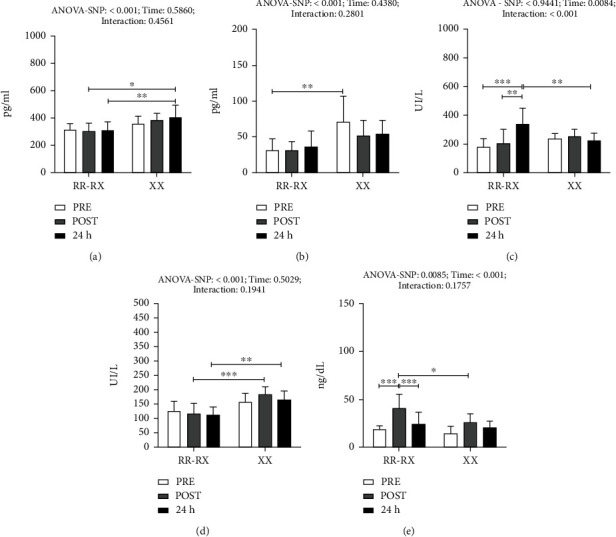
Serum concentration of indirect damage markers of muscle in genotype groups RR/RX and XX: (a) C-C motif chemokine ligand 2 (CCL2); (b) interleukin-8 (IL-8); (c) creatine kinase (CK); (d) lactate dehydrogenase (LDH); (e) myoglobin. White: pretraining blood marker values; gray: postexercise blood marker values (30 min after exercise); black: blood marker values 24 h after exercise. ^∗^*p* < 0.05; ^∗∗^*p* < 0.01; ^∗∗∗^*p* < 0.001.

Regarding the damage markers evaluated preexercise, postexercise, and 24 h after the training session, the effect of SNP was observed, showing increased CCL2 concentrations for the XX group in relation to the RR/RX group 24 h after the training session. Within the same group, no significant changes were observed regarding the time of collection, as shown in Figure 1(a).

IL-8 values also demonstrated an SNP effect. Increased values of this chemokine were observed in the XX group in the blood test taken before training in relation to the corresponding values of the RR/RX group taken at the same time. This suggests that the XX group already presented a more pronounced inflammatory profile in the pretraining situation, considering that they were men who actively exercised, which leads to the conclusion that there is a greater propensity to inflammation in carriers of the R allele (Figure 1(b)).

As for CK measurements, an interaction effect was observed, with higher increase concentrations in the RR/RX group at 24 h after training in relation to the XX group at the same evaluation moment. Within the same groups, only changes in the RR/RX group were observed, with a higher increase at 24 h after training in relation to the other moments evaluated.

The analysis also demonstrated an SNP effect for LDH, showing changes in the different periods analyzed. Higher LDH concentrations were identified in the XX group postexercise and after 24 h when compared to RR/RX in the same periods, as shown in Figure 1(d).

For myoglobin measurements, an SNP effect is observed in Figure 1(e), with higher values demonstrated in the RR/RX group postexercise in relation to the XX group at the same evaluated time. In addition, in the RR/RX group, a higher increase in myoglobin was also observed postexercise in relation to the other evaluated moments.

**Figure 2:**
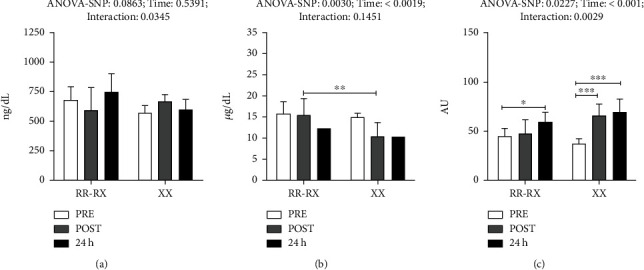
Hormonal parameters in genotype groups RR/RX and XX: (a) testosterone concentration, (b) cortisol concentration, and (c) T : C ratio. White: pretraining; gray: postexercise (30 min after exercise); black: 24 h after exercise. ^∗^*p* < 0.05; ^∗∗^*p* < 0.01; ^∗∗∗^*p* < 0.001.

In Figure 2(a), it can be seen that the results for SNP according to interaction and period for testosterone showed an interaction effect in both genotype groups, but the posttest values showed no differences.

The results for cortisol showed a reduction in the posttraining values in XX individuals when compared with RR/RX posttraining values (Figure 2(b)).

The effects analyzed for the testosterone/cortisol (T : C) ratio were observed by period and interaction. An increased T : C ratio was observed in RR/RX 24 h after exercise in relation to the pretraining ratio for this genotype group. A more pronounced increase was observed in XX posttraining and after 24 h in relation to the pretraining values (Figure 2(c)).

**Figure 3:**
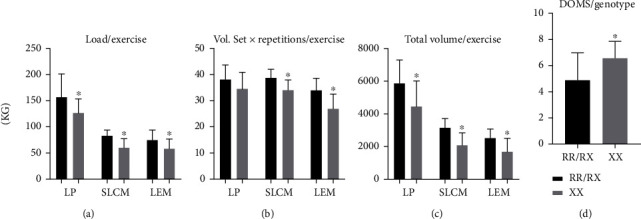
Comparison between load (a), volume of sets *x* repetitions (b), and total volume in each exercise (c), leg press (LP), seated leg curl machine (SLCM), leg extension machine (LEM), and delayed onset muscle soreness (DOMS) in groups RR/RX and XX (d). ^∗^*p* ≤ 0.05 for the difference between the RR/RX and XX groups.

In relation to the performance variables, an alteration was observed in the load used between the two groups for the LP, LEM, and SLCM, in which individuals with genetic profile XX presented lower strength values compared to the RR-RX group (Figure 3(a)).

In relation to the volume variables, there was a difference between the groups in the SLCM and LEM exercises: the XX group presented a worse performance compared to the RR/RX group (Figure 3(b)). Under the total volume, the XX group presented a lower performance than the RR-RX group in all three exercises of the study (Figure 3(c)). When analyzing DOMS, individuals with genetic profile XX presented higher values when compared to the RR/RX group (Figure 3(d)).

## 4. Discussion

The biochemical and performance marker results showed that the ACTN3-R577X polymorphism is associated with the inflammatory response and muscle damage after an acute ST session. In this study, higher CK, myoglobin, and cortisol concentrations indicated more elevated muscle damage in the RR/RX group. This can be explained by the higher volume of work performed by this group (Figure 3). The XX group presented a higher initial inflammatory profile and more intense DOMS even with lower volumes of training. It was expected that the XX group would present a greater inflammatory response, higher rates of muscular damage, and greater values in the scale of referred pain, thus requiring a longer period of recovery, but this expectation was not fully observed in the results.

As for the inflammatory response, a more pronounced inflammatory state was observed for the IL-8 pretest in the XX group. For CCL2, also in the XX group, there was a significant increase at 24 h after training. These findings are in agreement with those of a study conducted with soccer players who were evaluated immediately before and after an official match; higher values were observed in XX individuals before the acute stimulus when compared to the RR/RX group, which suggested an initial increased inflammatory response in physically active individuals [38]. On the other hand, other authors that also evaluated the inflammatory response after an acute training session did not find a difference between the evaluated posttest moments [40–42].

Regarding the response to muscle damage after an acute ST session until concentric failure, that is, with self-regulated workload, the RR-RX genotype presented higher values of muscle damage indicators. This can be explained by the greater workload of this group during training (Figure 3). In this respect, the present study demonstrated similarity with the results obtained by Coelho et al. [38] involving professional athletes submitted to an official soccer match. These results demonstrated the genotype profile effects, once the RR/RX group can perform a greater workload in the training, and as a consequence, this group would have more muscle damage.

On the other hand, after a maximal eccentric ST session, Broos et al. [21] showed a posttraining increase in muscle damage markers (CK and myoglobin), but without significant differences between the groups, probably due to the small sample sizes (four XX and four RR). However, a difference was observed between the reduction in maximum strength and pain reported postintervention, and the group deficient in alpha-actinin-3 (XX) presented higher scores in the subjective scale of posttraining pain, corroborating the present study. The authors suggested that this could be a protective mechanism against the development of injuries in subsequent eccentric exercises due to the greater stiffness exclusively of the type IIa muscle fibers observed in the RR group.

In another study with young people (10 XX and 9 RR) submitted to an eccentric ST session, Vincent et al. [43] demonstrated posttraining increases in muscle damage markers in both groups at the measured times. However, 24 h after the stimulus, the difference for the XX group was greater in relation to the RR group, a result that contradicts those of the present study. Like Broos et al. [21] and Vincent et al. [43], Venckunas et al. and Clarkson et al. [18, 44] did not find a difference in muscle damage markers after acute session training for the XX and RR groups. This supports the hypothesis that being a carrier of the gene encoding alpha-actinin-3 can be a protective mechanism against the development of injuries and muscle damage following an initial eccentric strength stimulus.

A probable explanation for the different results in different studies analyzing muscle damage through indirect markers, namely, CK, could be the training protocol proposed in each study. While the studies conducted by Clarkson et al. [44], Vincent et al. [43], and Venckunas et al. [18] presented identical training conditions applied to the groups, the present study limited the volume of exercise to concentric muscle failure. It can be inferred that individuals from the RR/RX group presented greater muscle damage when performing actions of greater magnitude of force [38]. Unlike the behavior of CK and myoglobin, the LDH data indicated a greater magnitude of muscle damage in the XX group. This can be considered a discrepant and complex finding to be explained, because the other data (CK, myoglobin, and cortisol) indicate that there was a greater level of demand for the RR/RX group.

Regarding the hormonal parameters (testosterone and cortisol), no relevant differences were observed between the collection times in the same group. A significant difference between the groups was only found for the posttraining cortisol concentration, with reduced values for the XX group in relation to the RR/RX group (Figure 2(b)). The analyses of these hormonal parameters are useful to determine the T : C ratio, which indicates a tendency to an anabolic or catabolic state. A catabolic condition previous to the training stimulus can be a sign of insufficient recovery from the previous stimulus or of some nutritional deficiency and can indicate overtraining [26, 27]. Although in the present study the T : C ratio showed greater levels in the XX group (Figure 2(c)), it is possible to interpret that the RR/RX group showed a greater catabolic tendency after the stimulus due to the higher levels of cortisol on this group. However, the analysis of the T : C ratio is only a tool, and it should not be analyzed in isolation to determine the overtraining status [45].

It was observed that despite the lower training volume of individuals with genotype XX, this group presented a greater inflammatory response and more referred pain in relation to the RR/RX genotypes. The present study demonstrated that the genotype XX presents greater concentrations of inflammation and DOMS markers, indicating the need for longer recovery periods, while the RR genotype can tolerate greater training loads, therefore presenting better absolute performance in strength and power activities.

A limitation of this study was the lack of standardization in relation to the participant diets, which may have influenced the results. There was also no long-term follow-up, which could have elucidated how these acute alterations favor chronic adaptations to exercise.

## 5. Conclusion

It is concluded that the *ACTN3* gene influences muscle damage and the inflammatory response resulting from an acute ST session. It was observed that after an acute ST session with a regulated high workload, the RR/RX genotype shows higher values of muscle damage markers and, consequently, a catabolic response, which can be explained by the higher workload of this group during exercise. On the other hand, genotype XX presents a basal inflammatory profile and greater DOMS responses after ST, albeit with lower performance values. The results from the present study can contribute to more individualized and optimized planning of the progression of training loads and recovery strategies for ST practitioners.

## Figures and Tables

**Table 1:** Characterization of the RR/RX and XX genotype groups regarding age, weight, height, body mass index (BMI), and % body fat.

	Age (years)	Weight (kg)	Height (m)	BMI (kg/m^2^)	% body fat
RR/RX (*n* = 18)	24.1 ± 3.8	78.1 ± 9.9	1.78 ± 0.1	24.7 ± 2.7	10.7 ± 5.3
XX (*n* = 9)	28.1 ± 3.9	78.6 ± 9.6	1.80 ± 0.1	25.4 ± 1.4	14.7 ± 4.6

## Data Availability

The authors confirm that the data supporting the findings of this study are available within the article.

## References

[1] Yang N., MacArthur D. G., Gulbin J. P. (2003). ACTN3 genotype is associated with human elite athletic performance. *American Journal of Human Genetics*.

[2] Ahmetov I. I., Egorova E. S., Gabdrakhmanova L. J., Fedotovskaya O. N. (2016). Genes and athletic performance: an update. *Medicine and Sport Science*.

[3] Baltazar-Martins G., Gutiérrez-Hellín J., Aguilar-Navarro M. (2020). Effect of ACTN3 genotype on sports performance, exercise-induced muscle damage, and injury epidemiology. *Sports*.

[4] Eynon N., Ruiz J. R., Femia P. (2012). The ACTN3 R577X polymorphism across three groups of elite male European athletes. *PLoS One*.

[5] Del Coso J., Hiam D., Houweling P., Pérez L. M., Eynon N., Lucía A. (2019). More than a ‘speed gene’: ACTN3 R577X genotype, trainability, muscle damage, and the risk for injuries. *European Journal of Applied Physiology*.

[6] Murtagh C. F., Brownlee T. E., Rienzi E. (2020). The genetic profile of elite youth soccer players and its association with power and speed depends on maturity status. *PLoS One*.

[7] Papadimitriou I. D., Lucia A., Pitsiladis Y. P. (2016). ACTN3 R577X and ACE I/D gene variants influence performance in elite sprinters: a multi-cohort study. *BMC Genomics*.

[8] Ma F., Yang Y., Li X. (2013). The association of sport performance with ACE and ACTN3 genetic polymorphisms: a systematic review and meta-analysis. *PLoS One*.

[9] Macarthur D. G., Seto J. T., Chan S. (2008). An Actn3 knockout mouse provides mechanistic insights into the association between *α*-actinin-3 deficiency and human athletic performance. *Human Molecular Genetics*.

[10] Pereira A., Costa A. M., Izquierdo M., Silva A. J., Bastos E., Marques M. C. (2013). ACE I/D and ACTN3 R/X polymorphisms as potential factors in modulating exercise-related phenotypes in older women in response to a muscle power training stimuli. *Age*.

[11] Romero-Blanco C., Artiga-González M. J., Gómez-Cabello A. (2020). Strength and endurance training in older women in relation to actn3 r577x and ace i/d polymorphisms. *International Journal of Environmental Research and Public Health*.

[12] Kittilsen H. T., Goleva-Fjellet S., Freberg B. I. (2021). Responses to maximal strength training in different age and gender groups. *Frontiers in Physiology*.

[13] MacArthur D. G., North K. N. (2004). A gene for speed? The evolution and function of *α*-actinin-3. *BioEssays*.

[14] Pimenta E. M., Coelho D. B., Cruz I. R. (2012). The ACTN3 genotype in soccer players in response to acute eccentric training. *European Journal of Applied Physiology*.

[15] Massidda M., Voisin S., Culigioni C. (2019). ACTN3 R577X polymorphism is associated with the incidence and severity of injuries in professional football players. *Clinical Journal of Sport Medicine*.

[16] Clos E., Pruna R., Lundblad M., Artells R., Esquirol Caussa J. (2019). ACTN3 single nucleotide polymorphism is associated with non-contact musculoskeletal soft-tissue injury incidence in elite professional football players. *Knee Surgery, Sports Traumatology, Arthroscopy*.

[17] Pickering C., Kiely J. (2017). ACTN3: more than just a gene for speed. *Frontiers in Physiology*.

[18] Venckunas T., Skurvydas A., Brazaitis M., Kamandulis S., Snieckus A., Moran C. N. (2012). Human alpha-actinin-3 genotype association with exercise-induced muscle damage and the repeated-bout effect. *Applied Physiology, Nutrition, and Metabolism*.

[19] Del Coso J., Valero M., Salinero J. J. (2017). ACTN3 genotype influences exercise-induced muscle damage during a marathon competition. *European Journal of Applied Physiology*.

[20] Seto J. T., Lek M., Quinlan K. G. R. (2011). Deficiency of *α*-actinin-3 is associated with increased susceptibility to contraction-induced damage and skeletal muscle remodeling. *Human Molecular Genetics*.

[21] Broos S., Malisoux L., Theisen D. (2019). The stiffness response of type IIa fibres after eccentric exercise-induced muscle damage is dependent on ACTN3 r577X polymorphism. *European Journal of Sport Science*.

[22] Gentil P., Pereira R. W., Leite T. K. M., Bottaro M. (2011). ACTN3 R577X polymorphism and neuromuscular response to resistance training. *Journal of Sports Science & Medicine*.

[23] Paulsen G., Mikkelsen U. R., Raastad T., Peake J. M. (2012). Leucocytes, cytokines and satellite cells: what role do they play in muscle damage and regeneration following eccentric exercise?. *Exercise Immunology Review*.

[24] Peake J. M., Neubauer O., Gatta P. A. D., Nosaka K. (2017). Muscle damage and inflammation during recovery from exercise. *Journal of Applied Physiology*.

[25] Baumert P., Lake M. J., Stewart C. E., Drust B., Erskine R. M. (2016). Genetic variation and exercise-induced muscle damage: implications for athletic performance, injury and ageing. *European Journal of Applied Physiology*.

[26] Uchida M. C., Pereira Bacurau R. F., Navarro F. (2004). Alteração da relação testosterona: cortisol induzida pelo treinamento de força em mulheres. *Revista Brasileira de Medicina do Esporte*.

[27] Fry A. C., Kraemer W. J., Ramsey L. T. (1998). Pituitary-adrenal-gonadal responses to high-intensity resistance exercise overtraining. *Journal of Applied Physiology*.

[28] Shang X., Li Z., Cao X. (2015). The association between the ACTN3 R577X polymorphism and noncontact acute ankle sprains. *Journal of Sports Sciences*.

[29] Kim J. H., Jung E. S., Kim C.-H., Youn H., Kim H. R. (2014). Genetic associations of body composition, flexibility and injury risk with ACE, ACTN3 and COL5A1 polymorphisms in Korean ballerinas. *Journal of Exercise Nutrition and Biochemistry*.

[30] Miot H. A. (2011). Tamanho da amostra em estudos clínicos e experimentais. *Jornal Vascular Brasileiro*.

[31] Simão R., Farinatti P. D., Polito M. D., Maior A. S., Fleck S. J. (2005). Influence of exercise order on the number of repetitions performed and perceived exertion during resistance exercises. *Journal of Strength and Conditioning Research*.

[32] Helms E., Cronin J., Storey A., Zourdos M. (2016). Application of the repetitions in reserve-based rating of perceived exertion scale for resistance training. *Strength and Conditioning Journal*.

[33] Schoenfeld B., Grgic J. (2018). Evidence-based guidelines for resistance training volume to maximize muscle hypertrophy. *Strength and Conditioning Journal*.

[34] Diniz R. C. R., Martins-Costa H. C., Machado S. C., Lima F. V., Chagas M. H. (2014). Repetition duration influences ratings of perceived exertion. *Perceptual and Motor Skills*.

[35] Sampson J. A., Donohoe A., Groeller H. (2014). Effect of concentric and eccentric velocity during heavy-load non-ballistic elbow flexion resistance exercise. *Journal of Science and Medicine in Sport*.

[36] Goto K., Nagasawa M., Yanagisawa O., Kizuka T., Ishii N., Takamatsu K. (2004). Muscular adaptations to combinations of high- and low-intensity resistance exercises. *Journal of Strength & Conditioning Research*.

[37] Miller S. A., Gallie D. R., Sleat D. E., Watts J. W., Turner P. C., Wilson T. M. (1988). Mutational analysis of the tobacco mosaic virus 5′-leader for altered ability to enhance translation. *Nucleic Acids Research*.

[38] Coelho D. B., Pimenta E. M., Rosse I. C. (2019). Alpha-actinin-3 R577X polymorphism influences muscle damage and hormonal responses after a soccer game. *Journal of Strength and Conditioning Research*.

[39] Ohnhaus E. E., Adler R. (1975). Methodological problems in the measurement of pain: a comparison between the verbal rating scale and the visual analogue scale. *Pain*.

[40] Hirose L., Nosaka K., Newton M. (2004). Changes in inflammatory mediators following eccentric exercise of the elbow flexors. *Exercise Immunology Review*.

[41] Ross M. L. R., Halson S. L., Suzuki K. (2010). Cytokine responses to carbohydrate ingestion during recovery from exercise-induced muscle injury. *Journal of Interferon & Cytokine Research*.

[42] Paulsen G., Benestad H. B., Strøm-Gundersen I., Mørkrid L., Lappegård K. T., Raastad T. (2005). Delayed leukocytosis and cytokine response to high-force eccentric exercise. *Medicine and Science in Sports and Exercise*.

[43] Vincent B., Windelinckx A., Nielens H. (2010). Protective role of *α*-actinin-3 in the response to an acute eccentric exercise bout. *Journal of Applied Physiology*.

[44] Clarkson P. M., Hoffman E. P., Zambraski E. (2005). ACTN3 and MLCK genotype associations with exertional muscle damage. *Journal of Applied Physiology*.

[45] Grandou C., Wallace L., Impellizzeri F. M., Allen N. G., Coutts A. J. (2020). Overtraining in resistance exercise: an exploratory systematic review and methodological appraisal of the literature. *Sports Medicine*.

